# Balancing risks of recurrent venous thromboembolism and bleeding with extended anticoagulation: a decision analysis

**DOI:** 10.1016/j.rpth.2023.102274

**Published:** 2023-11-26

**Authors:** Maria A. de Winter, Kednapa Thavorn, Steven H.J. Hageman, Mathilde Nijkeuter, Philip S. Wells

**Affiliations:** 1 Department of Acute Internal Medicine, University Medical Center Utrecht, Utrecht, The Netherlands; 2 Department of Internal Medicine, Diakonessenhuis, Utrecht, The Netherlands; 3 School of Epidemiology and Public Health, University of Ottawa and the Ottawa Hospital Research Institute, Ottawa, Ontario, Canada; 4 Department of Vascular Medicine, University Medical Center Utrecht, Utrecht, The Netherlands

**Keywords:** anticoagulation, bleeding, quality-adjusted life years, risk, venous thromboembolism

## Abstract

**Background:**

A decision to stop or continue anticoagulation after 3 months of anticoagulation for venous thromboembolism (VTE) should be made by weighing individual risks of recurrence and bleeding.

**Objectives:**

To determine the optimal ratio of recurrence risk reduction to increase the risk of bleeding in terms of maximizing quality-adjusted life years (QALYs) gained.

**Methods:**

Using a microsimulation model, outcomes within 5 years were simulated after assigning extended treatment if absolute recurrence risk reduction outweighed absolute increase in clinically relevant bleeding risk (International Society on Thrombosis and Haemostasis definition), weighted by a certain ratio. Data were simulated based on the Bleeding Risk Study, a prospective cohort including patients after ≥3 months of anticoagulation for unprovoked VTE or provoked VTE with history of VTE. The VTE-PREDICT risk score was used to estimate 5-year risks of recurrent VTE and clinically relevant bleeding.

**Results:**

Among 10,000 individuals (mean age, 60.2 years, 36% female), the ratio of 0.90 (95% CI, 0.51-3.40; ie, bleeding is considered 0.90 the severity of recurrent VTE), with 99% of patients assigned extended anticoagulation, was considered optimal and resulted in 93 (95% CI, −23 to 203) additional QALYs compared with the least favorable ratio (5.10, 0% extended anticoagulation). At the optimal ratio, treatment based on VTE-PREDICT yielded 44 (95% CI, −69 to 157) additional QALYs versus standard of care.

**Conclusion:**

With the current evidence, the optimal ratio between relevant bleeding risk and absolute recurrence risk reduction remains uncertain. Our results confirm that clinical equipoise exists regarding the decision to stop or continue anticoagulation after initial VTE treatment, emphasizing the importance of shared decision-making.

## Essentials

• The duration of the treatment for venous thrombosis is decided by weighing the risks of recurrence and bleeding.
• This decision analysis investigated the optimal weighing ratio between these individual risks.
• Optimal and least optimal ratios (99% vs 0% extended treatment) differed by <1 day in perfect health.
• Shared decision-making is essential when deciding to stop or continue anticoagulation.

## 1 Introduction

After at least 3 months of anticoagulation for venous thromboembolism (VTE), treatment may be stopped or continued indefinitely [[1], [2], [3]]. When treatment is stopped, risk of recurrent VTE may be as high as 25% in 5 years [4]. Anticoagulant treatment is highly effective at preventing VTE recurrence but carries a 1% to 2% annual risk of major bleeding [1]. Therefore, guidelines advise deciding to stop or continue anticoagulation by carefully weighing risk of recurrent VTE and risk of bleeding with consideration of patient’s preferences [[1], [2], [3]]. For current practice, this resulted in the recommendation to stop anticoagulation after 3 months for VTE provoked by major transient risk factors (eg, major surgery) and to at least consider continuing treatment for all other patients in the absence of a high bleeding risk. However, this approach does not adequately capture the heterogeneity of risks.

To facilitate individualized treatment decisions, the VTE-PREDICT risk score has been developed to predict individual patients’ absolute risks of recurrent VTE and clinically relevant bleeding (International Society on Thrombosis and Haemostasis [ISTH] definition) with and without extended anticoagulation [5]. External validation in multiple datasets has pointed out that the model may be used to adequately predict risks of recurrent VTE and clinically relevant bleeding, and an online calculator is now available. Risks and treatment benefits can be discussed by physicians and their patients in a process of shared decision-making to decide on the best treatment for an individual. Improved patient involvement and shared decision-making may increase patient satisfaction with care and treatment adherence while reducing costs [6].

However, how to weigh absolute risks is currently undecided. Several aspects should be considered, such as case fatality rates, risk of long-term complications, costs, and patients’ preferences. Furthermore, interpreting risks may be challenging for patients and physicians [7]. The most obvious approach would be to directly compare absolute recurrence risk reduction and increase in risk of bleeding with extended anticoagulation. However, it is unlikely that patients and physicians will consider these events to be equivalent, and this approach does not capture consequences in terms of quality of life. Alternatively, treatment could be initiated starting from a certain ratio in which treatment benefits outweigh treatment harms, but such data does not exist.

Therefore, the aims of this study were:

1. To determine the optimal ratio of VTE recurrence risk reduction to increase the risk of clinically relevant bleeding (ISTH definition) with extended anticoagulation based on a composite metric that incorporates patient preference and life expectancy, namely quality-adjusted life years (QALYs) gained, to inform the decision on when to stop or continue anticoagulation after the initial treatment for VTE on a group level; and
2. To evaluate the number of bleeding and recurrent VTE events, fatalities, and QALYs achieved by following existing clinical guidelines versus using the VTE-PREDICT risk score with the optimal ratio determined in aim 1 for treatment decisions after initial treatment for VTE.

## 2 Methods

### 2.1 Study population

The study population consisted of adult patients with VTE who completed an initial anticoagulant treatment of at least 3 months, representative of the total VTE population, with available baseline data on type and duration of anticoagulation and most predictors included in the VTE-PREDICT risk score (Supplementary Table S1).

### 2.2 Data sources

The dataset used for the analysis was simulated based on data from the Bleeding Risk Study [8]. This was a multicenter, multinational prospective cohort study with the aim of developing a new prediction tool for major bleeding. Patients who completed at least 3 months of oral anticoagulation for unprovoked VTE or provoked VTE with a history of VTE were included. A more detailed description of this study has been published elsewhere [8]. The study complied with the Declaration of Helsinki. Ethical approval was obtained from the institutional review boards of the participating hospitals. Informed consent was provided by all patients.

Prior to simulating the data, single imputation using predictive mean matching was used to impute sporadically missing values in the Bleeding Risk Study data (body mass index <1%, hemoglobin 1%, estimated glomerular filtration rate [eGFR] 7%). Variables not available in the Bleeding Risk Study were sampled, depending on availability, based on values in the REVERSE I and REVERSE II studies (VTE associated with estrogen therapy), Canadian population values (systolic blood pressure), or the VTE-PREDICT study population (platelet count) [5,[9], [10], [11]]. History of cancer, liver disease, alcohol abuse, and recent surgery was assumed to be absent for all patients as a high bleeding risk, and active cancer was exclusion criteria for the Bleeding Risk Study; pregnancy as provoking factor was assumed to be absent for all patients given the proportion of female patients and age range of the study population.

### 2.3 Outcomes

Outcomes of interest were recurrent VTE, clinically relevant bleeding, all-cause mortality, and QALYs. Recurrent VTE was defined as objectively confirmed deep venous thrombosis (DVT) and/or pulmonary embolism. Clinically relevant bleeding is defined as a composite of clinically relevant nonmajor and major bleeding according to the ISTH definitions [12,13]. QALY is the number of years individuals live multiplied by how much they value a certain level of health (health utility, ranging from 0 to 1) each year.

### 2.4 Estimating the individual effect of extended anticoagulant treatment

For every individual patient, the VTE-PREDICT risk score was used to calculate absolute recurrence risk reduction and increase in the risk of clinically relevant bleeding with extended anticoagulation within 5 years. A detailed description of the methodology and results of VTE-PREDICT has been published elsewhere [5]. For the base case analysis, a pooled estimate for the effect of extended treatment with full-dose direct oral anticoagulant (DOAC) was used (Supplementary Table S2).

### 2.5 Treatment decision algorithm

Clinical decision-making was simulated based on predicted absolute treatment effects and current guidelines.

The following hypothetical scenarios were investigated:

• All ratios between absolute recurrence risk reduction and increase in risk of clinically relevant bleeding between 10:1 and 1:10 were considered for the base case analysis. Extended anticoagulation was assigned when predicted recurrence risk reduction outweighed the predicted risk of bleeding, adjusted by a certain ratio. This ratio was defined as the relative relevance of a single recurrent VTE versus a single clinically relevant bleeding event and was implemented mathematically by multiplying the bleeding risk increase with the ratio. From a certain ratio onwards, all or no patients were assigned extended anticoagulation. Ratios below the maximum ratio for which all patients were assigned extended treatment and ratios above the minimum ratio for which no patients were assigned extended treatment were excluded.
• Current standard of care according to international guidelines, ie, extended treatment for all patients with unprovoked VTE or VTE provoked by minor risk factors or a history of VTE not being provoked by major transient risk factors in the absence of a high bleeding risk [[1], [2], [3]]. To identify patients at high risk of bleeding, the American College of Chest Physicians score was used, as this score is frequently included in national and international guidelines [2].

### 2.6 Model description

**Figure 1:**
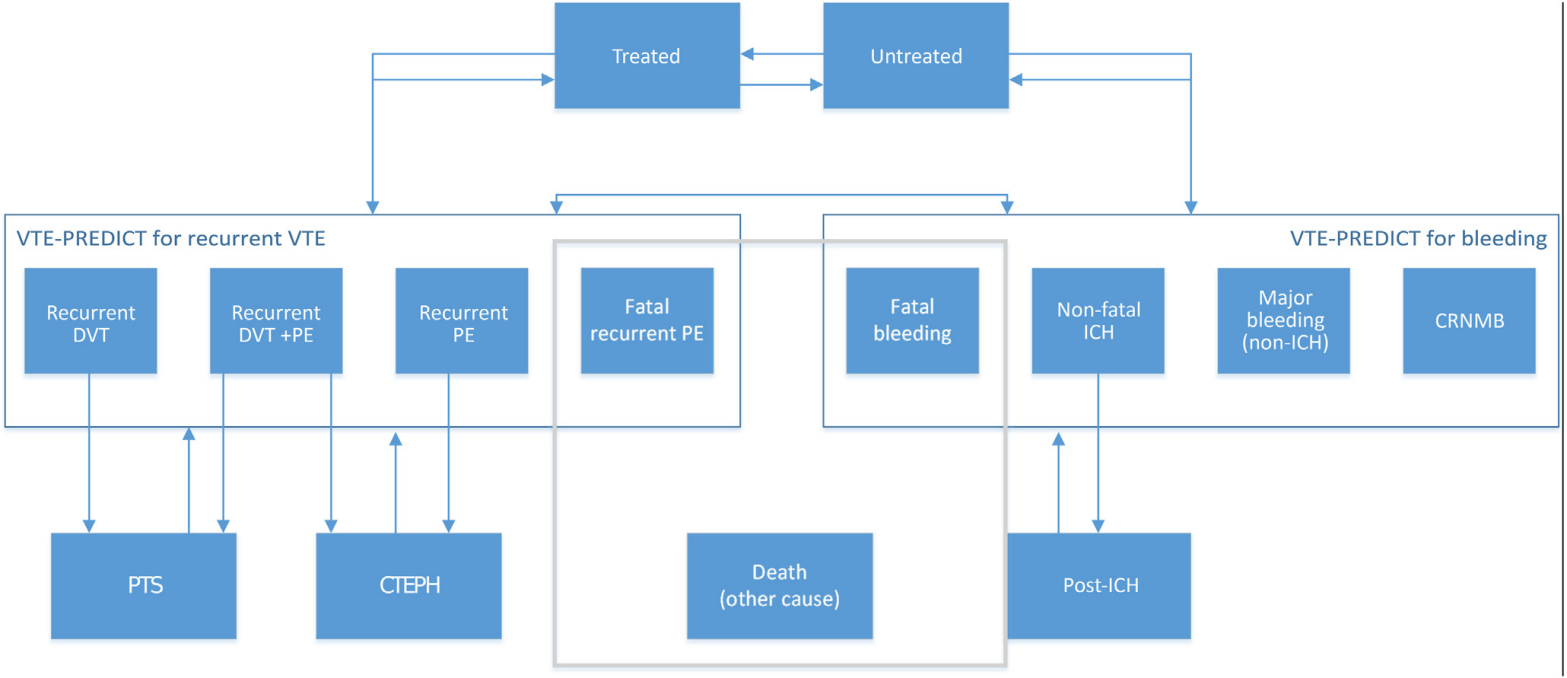
Simplified model schematic illustrating states and probabilities. CRNMB, clinically relevant, nonmajor bleeding; CTEPH, chronic thromboembolic pulmonary hypertension; DVT, deep venous thrombosis; ICH, intracranial hemorrhage; PE, pulmonary embolism; PTS, postthrombotic syndrome; VTE, venous thromboembolism.

A microsimulation model was developed to simulate outcomes of each scenario in terms of the number of events (recurrence and clinically relevant bleeding [ISTH definition]) and all-cause mortality within 5 years. A microsimulation model starts with a group of virtual patients, each of whom is different, and models how their disease and treatment develop over time. The follow-up time is divided into 3-month cycles. At the end of each cycle, patients could remain in the same health state (eg, healthy, bleeding event, and recurrent VTE) or progress to a different health state. Patients transition across health states based on individual transition probabilities. Individual transition probabilities are among others, based on patient characteristics (medical history, risk factors), patients’ previous health status, and treatment. These models can be used to simulate the impact of interventions in a population. The model is useful for prediction of effectiveness of different management strategies [14]. Possible states and transitions are illustrated in Figure 1. Individual transition probabilities are calculated based on individual estimates of risks of recurrent VTE, clinically relevant bleeding and mortality, and overall probabilities available from previous literature. Risks of recurrent VTE and bleeding for each patient were calculated using 5-year estimates from the VTE-PREDICT risk score. We used a Weibull model to estimate 5-year risk of mortality developed in the original Bleeding Risk Study data (Supplementary Table S3) [[15], [16], [17]]. All health states and (dis)utilities included are shown in Supplementary Table S4. Utilities indicate how much individuals value their well-being associated with being in a certain health state (range, 0-1). Disutilities represent the negative impact that a health condition imposes on an individual’s well-being. Both utilities and disutilities are used to adjust the remaining life years of individuals to their associated quality of life. An overview of all other model assumptions is provided in Supplementary Table S5.

### 2.7 Determining the optimal ratio

The proportion of patients with extended anticoagulant treatment, type and number of events, and total number of QALYs with each ratio between recurrence risk reduction and increase in risk of bleeding were assessed. The ratio resulting in the highest number of QALYs within 5 years was considered the optimal ratio. Probabilistic analyses were performed to assess robustness of the results, for which the microsimulation model was repeated 1000 times with probabilities and utilities randomly sampled from assumed underlying distributions, including binomial, normal, and gamma distributions, using Monte Carlo simulations. The final estimate of the optimal ratio and CIs were based on probabilistic analyses (mean value of simulations).

### 2.8 Comparison with standard of care

Subsequently, proportion of patients with treatment, number of outcome events, and number of QALYs obtained with standard of care according to guidelines ie, extended anticoagulation for patients with unprovoked VTE without a high risk of bleeding (American College of Chest Physicians score ≥2 [[1], [2], [3]]) was simulated. Results were compared to results obtained when treating patients according to the optimal ratio. Characteristics of patients with and without extended treatment according to both scenarios in simulated data were compared.

### 2.9 Sensitivity analyses

One-way sensitivity analyses were performed for all utility values and treatment effect estimates. Scenario analyses were based on probabilistic analyses to assess the impact of changing model assumptions, shown in Supplementary Table S6. Due to clear convergence in the base case analyses before 400 repetitions, all scenario analyses were only simulated 400 times (Supplementary Figure S4).

All analyses were performed using R Statistical Software, version 4.0.3.

## 3 Results

Data from 2546 patients in the Bleeding Risk Study were used to construct a dataset for analysis. The baseline characteristics of the original study population and 10,000 simulated cases used for the present analysis were well-aligned (Table 1). In the simulated dataset, mean age was 60.2 years (SD, 14.3), 36% were female, and 89% had unprovoked VTE. The distribution of predicted 5-year risks of recurrent VTE and bleeding according to the VTE-PREDICT risk score in this population is shown graphically in Supplementary Figure S1. The median untreated 5-year risk of recurrent VTE was 8.9% (range, 4.6%-13.4%; IQR, 7.8-9.6), whereas median risk of clinically relevant bleeding was 3.4% (range, 1.1%-6.1%; IQR, 2.8-4.2).

**Table 1:** Baseline characteristics of the Bleeding Risk Study population and the simulated dataset used for analysis.

	Bleeding Risk Study, *n* (%)^a^	Simulated dataset for analysis, *n* (%)^a^
	*n* = 2546	*N* = 10,000
Female sex	918 (36)	3554 (36)
Age (years), mean (SD)	60.1 (14.7)	60.2 (14.3)
Current smoker	291 (11)	1137 (11)
**Medical history**		
Prior stroke	83 (3)	307 (3)
History of bleeding	84 (3)	316 (3)
History of VTE	926 (36)	3614 (36)
History of cancer	n.a.	0 (0)
Atherosclerotic cardiovascular disease	128 (5)	511 (5)
Diabetes mellitus	279 (11)	1078 (11)
**Index event**		
PE with or without DVT	1226 (48)	4806 (48)
Distal DVT only	11 (1)	n.a.
Provoked by estrogen therapy	n.a.	601 (6)
Provoked by surgery, trauma, or immobilization	149 (6)	571 (6)
Unprovoked VTE	n.a.	8862 (89)
**Physical examination and laboratory measurements**		
BMI (kg/m^2^)	31.2 (7.2)	31.0 (6.7)
Systolic blood pressure (mm Hg)	n.a.	118.0 (16.3)
Hemoglobin (g/dL)	14.1 (1.8)	14.2 (1.5)
eGFR (mL/min)	82.2 (22.4)	82.6 (21.0)
Platelet count (x 10^9^/L)	n.a.	240.5 (65.2)
**Concomitant treatment**		
Antiplatelet therapy	150 (6)	544 (5)
NSAIDs	97 (4)	346 (3)

BMI, body mass index; DVT, deep venous thrombosis; eGFR, estimated glomerular filtration rate; Hb, hemoglobin; PE, pulmonary embolism; n.a., not applicable; NSAIDs, nonsteroidal anti-inflammatory drugs; VTE, venous thromboembolism.

a Unless otherwise specified.

### 3.1 Results of microsimulation model

**Figure 2:**
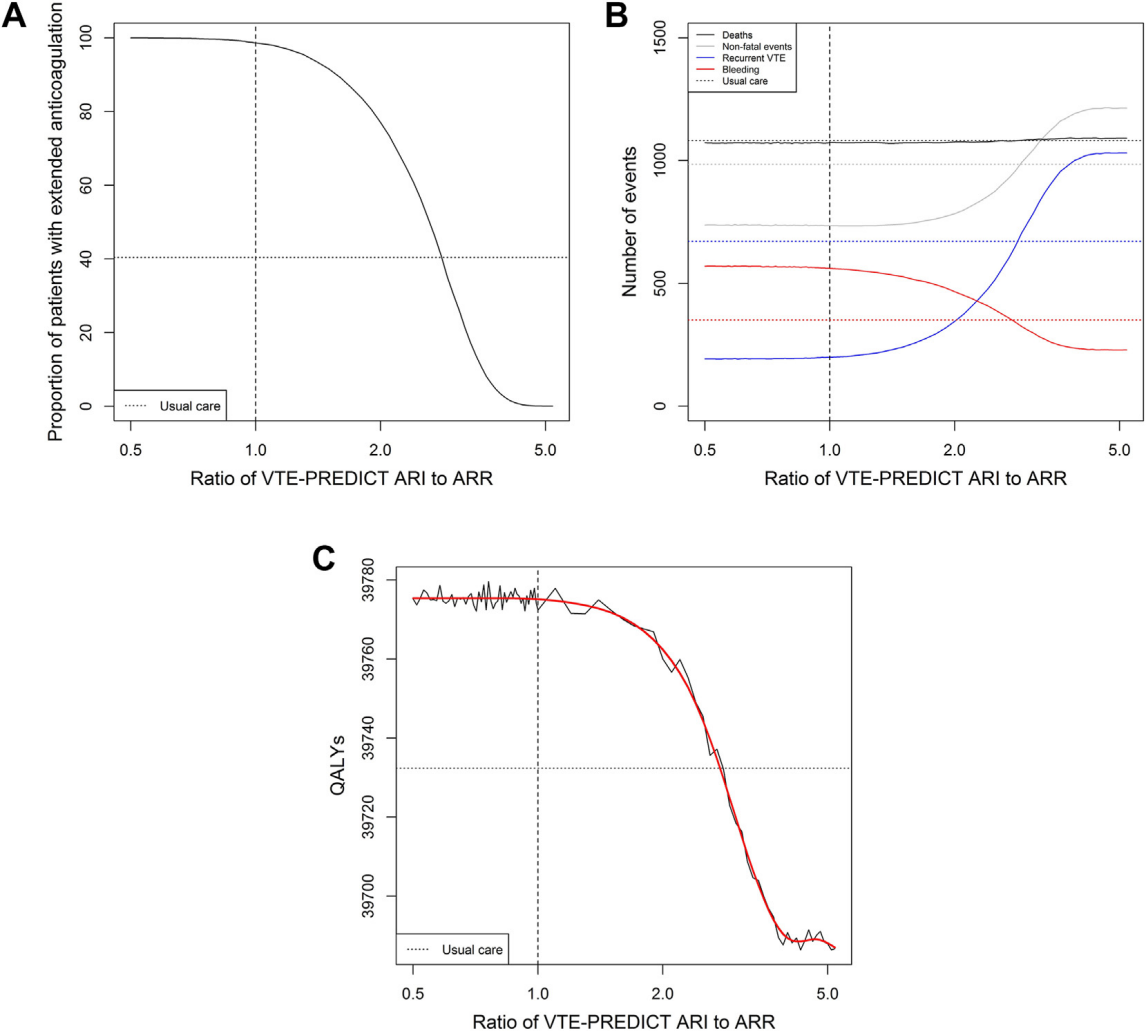
Treatment assignment (A), number of events (B), and number of quality-adjusted life years (C) per 10,000 cases within 5 years for each of the assessed ratios between recurrence risk reduction and increase in risk of bleeding with VTE-PREDICT and usual care. Figures are based on probabilistic analysis using 1000 Monte Carlo simulations. ARI, absolute risk increase; ARR, absolute risk reduction; QALY, quality-adjusted life year.

**Figure 3:**
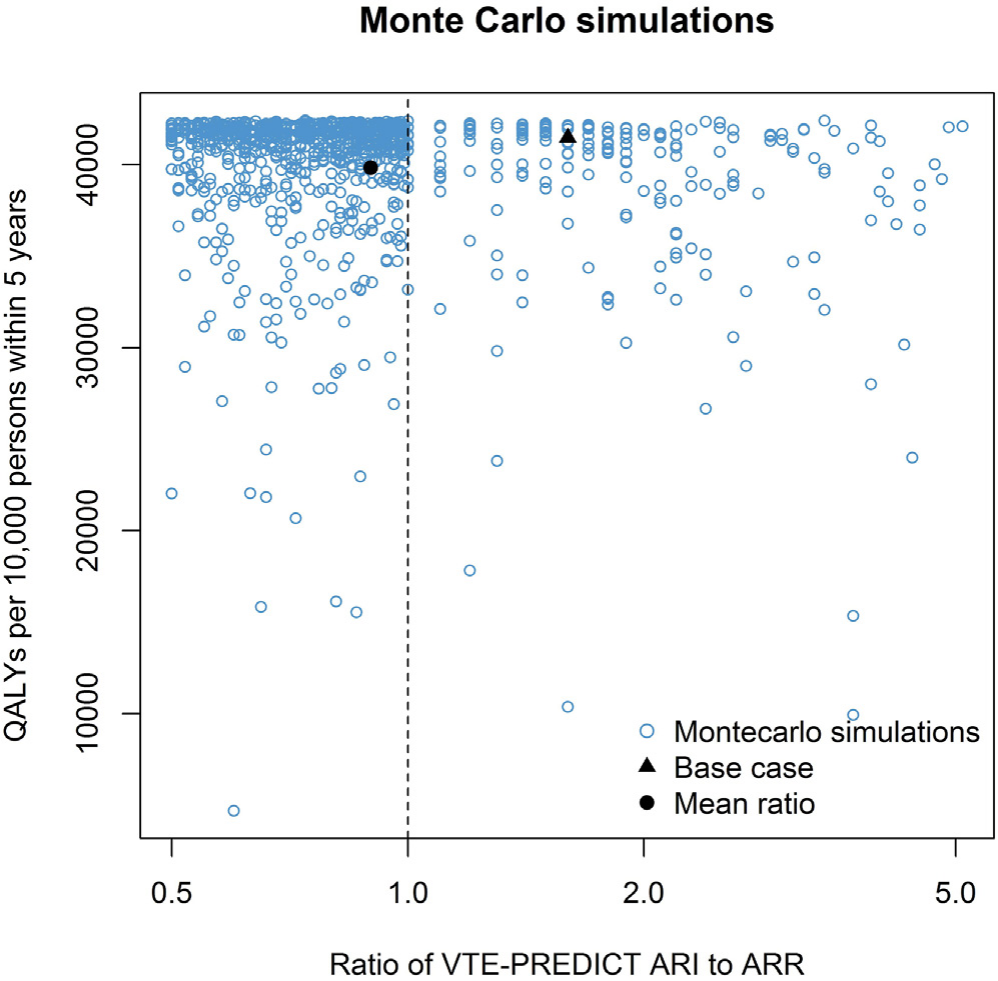
Probabilistic analyses. Optimal ratio and number of quality-adjusted life years (QALYs) associated with the optimal ratio in each of the Monte Carlo simulations (*n* = 1000). ARI**,** absolute risk increase; ARR**,** absolute risk reduction.

The ratio between recurrence risk reduction and bleeding risk increase leading to the highest number of QALYs was 0.90 (95% CI, 0.51-3.40). With this ratio, the severity of a clinically relevant bleeding event is considered 90% of the severity of a recurrent VTE event. In this scenario, 99.3% of the patients were assigned extended anticoagulation. The optimal ratio resulted in an additional 93 QALYs (95% CI, -23 to 203, with 339 additional clinically relevant bleeding events, 837 fewer VTE recurrences, and 21 fewer deaths) among 10,000 patients within 5 years of follow-up compared with the least favorable ratio (5.10). For ratios of 0.50 and below, all patients were assigned to extended anticoagulation; for ratios of 5.20 and above, no patients were to receive extended anticoagulation. With increasing ratios, more weight is assigned to bleeding, and proportion of patients on extended treatment decreases (Figure 2). This is associated with an increase in VTE recurrences and a decrease in clinically relevant bleeding events. The number of fatal events increased from 1070 to 1092. The lowest number of acute events occurred at a ratio of 1.10, whereas the lowest total number of deaths occurred at a ratio of 0.72. Results of the base case analysis, using point estimates of all parameters in a single run, are shown in Supplementary Figure S2. Although the overall conclusions were consistent with the probabilistic analysis, the point estimate of the optimal ratio obtained from the deterministic analysis was higher (1.60), likely reflecting random variation, as can be appreciated from Supplementary Figure S2C and Figure 3. Supplementary Figure S4 indicates that outcome estimates remained stable after approximately 300 repetitions, indicating adequate convergence.

### 3.2 Comparison with standard of care

Comparing the VTE-PREDICT strategy with the newly defined optimal ratio (0.90) to the current standard of care, more QALY gains (+44, 95% CI, -69 to 157), more patients being assigned extended anticoagulation (99% vs 40%), less VTE recurrences, and more clinically relevant bleeding events were found (Table 2). When comparing the standard of care to other ratios, the number of acute and fatal events was higher in the standard of care scenario than with VTE-PREDICT, with ratios of 2.9 and lower. Similarly, treatment according to VTE-PREDICT resulted in more QALYs compared with treatment according to standard of care for ratios up to 3.3 (Figure 2). Characteristics of patients with and without extended anticoagulation according to VTE-PREDICT using the optimal ratio and according to usual care are shown in Supplementary Table S7.

**Table 2:** Treatment assignment, clinical outcomes, and quality-adjusted life years within 5 years per 10,000 patients following treatment decisions based on the optimal ratio versus standard of care.

	Standard of care (95% CI)	Optimal ratio of 0.90 (95% CI)	Difference *n* (%)	*P* value for difference
Extended anticoagulation	4044	9932	+5888 (+146)	n.a.
Recurrent VTE	670 (290-1052)	197 (58-336)	-473 (-71)	<.001
Clinically relevant bleeding	352 (102-603)	567 (52-1081)	+215 (+61)	<.001
Death	1081 (54-4991)	1072 (44-4843)	20 (-2)	.745
QALYs	39,732 (31,761-47,704)	39,776 (31,782-47,770)	+44 (+1)	.811

QALY, quality-adjusted life year; VTE venous thromboembolism.

### 3.3 Sensitivity analyses

Results of 1-way sensitivity analyses, in which one parameter was changed at a time, are shown graphically in Supplementary Figure S3. When varying treatment efficacy and/or safety, the optimal ratio changed, with a very modest difference in total QALYs (<0.1%). Health utilities for both acute events and chronic complications (ie, chronic thromboembolic pulmonary hypertension, postthrombotic syndrome, postintracranial hemorrhage state) had a very minor impact on total number of QALYs, and the impact did not meaningfully affect the optimal ratio.

### 3.4 Scenario analyses

When assessing the effect of the reduced rather than full-dose DOAC scenario (Supplementary Figure S5A), possible range of ratios varied from 3.90 (all patients on extended treatment) to 42.30 (none on extended treatment). There was a low number of clinically relevant bleeding events, with limited variation across ratios. This resulted in a lower number of fatal and nonfatal events and a higher number of QALYs. The optimal ratio in this scenario was 10.15 (95% CI, 3.66-28.18). Compared to the optimal ratio with full-dose DOAC, this resulted in 126 additional QALYs, 98 additional VTE recurrences, 324 less clinically relevant bleeding events, and 58 fewer deaths. For the other scenarios, possible range of ratios was equal to the base case analysis. Considering treatment discontinuation unrelated to bleeding events (Supplementary Figure S5A), fewer bleeding events and more recurrent VTE events were observed, with an optimal ratio of 0.94 (95% CI, 0.51-3.80). When applying a disutility for anticoagulant treatment (Supplementary Figure S5C), higher ratios, resulting in a lower proportion of patients on extended treatment, yielded the most QALYs (optimal ratio, 4.39; 95% CI, 3.20-5.30). When applying the same death rate for all patients (Supplementary Figure S5D), number of deaths was considerably lower, resulting in more QALYs and an optimal ratio of 0.82 (95% CI, 0.52-1.91).

## 4 Discussion

A ratio between recurrence risk reduction and increase in risk of bleeding with extended treatment with full-dose DOAC of 0.90 (95% CI, 0.51-3.40), with 99% of patients assigned to extended anticoagulation, was found to result in the highest number of QALYs within 5 years of follow-up in 10,000 simulated cases. Weighing bleeding risks more heavily than recurrence risks (higher ratios) resulted in a decreasing number of patients on extended anticoagulation, and with that, more VTE recurrences, less clinically relevant bleeding events, and a marginally higher number of deaths.

The wide CI indicates great uncertainty around the point estimate for the optimal ratio. The proportion of patients who received extended anticoagulation according to all ratios within the interval ranged from hardly any to almost all patients. Several factors may have contributed to the great extent of variation observed. First, sum of fatal and nonfatal events was comparable for each of the ratios. Across ratios, recurrent VTE events are merely exchanged for clinically relevant bleeding events when increasing the proportion of patients on extended anticoagulation. As a result, difference in sum of QALYs across ratios was very small (<1% variation). Second, many different parameters were included in the microsimulation model, all inducing variation. As can be appreciated from scenario and sensitivity analyses, treatment effect estimates were found to be especially influential.

We found a marginal difference in QALYs on a group level when deciding on treatment with the VTE-PREDICT risk score using the optimal ratio versus the least favorable ratio. The difference of 93 QALYs per 10,000 patients within 5 years corresponds to 0.68 days in perfect health per person per year. For a gain of less than 1 day in perfect health, 99% of all patients need to be on extended anticoagulation for a year. Anticoagulant treatment involves costs and may cause side effects other than bleeding. Although some patients experience comfort and a feeling of being protected while on anticoagulation, others may consider using medication to be burdensome or stigmatizing [18]. This was modeled in a scenario analysis as a disutility of 0.99 per 3-month cycle. Unsurprisingly, taking this into account, a ratio in which <1% of patients were to be treated was found optimal in this situation. Our findings were confirmed by a recently published cost-effectiveness analysis in patients with a first unprovoked VTE, in which indefinite anticoagulation was found to result in -0.075 QALYs per person. Moreover, indefinite anticoagulation costs $16,014 more per person per year, while it did not result in any benefit in terms of QALYs [19].

Whereas the limited gain in QALYs and associated costs when treating every patient with extended anticoagulation would suggest a preference for short-term treatment, guidelines generally focus on continuing treatment unless contraindications exist. Moreover, previous studies have shown that patients, as well as physicians, tend to put most emphasis on preventing recurrent VTE as risk of bleeding may be difficult to grasp. Both are sometimes willing to accept very high risks of bleeding to prevent VTE recurrence. Another potential explanation for extended anticoagulation often being considered treatment of choice could be the definition of bleeding generally used in previous literature. Traditionally, researchers and clinicians focused on major bleeding rather than the composite of clinically relevant, nonmajor bleeding and major bleeding, which was used for the VTE-PREDICT risk score. However, clinically relevant, nonmajor bleeding is also associated with healthcare costs and reduced quality of life [[20], [21], [22]]. As major bleeding is only a small proportion of all clinically relevant bleeding events, and risks are generally lower than for recurrent VTE, this may have led to a different perspective on the balance between risks of recurrent VTE and bleeding. Future economic evaluations should be conducted to account for both costs and benefits of extended anticoagulation.

Comparing the VTE-PREDICT risk score to standard of care according to guidelines, we found that total number of QALYs was higher for all ratios up to 3.3. On a group level, using estimates based on the total population, treating all versus treating none has a relatively limited impact on QALYs. However, for individual patients, treatment may have a profound impact on quality of life [18]. Individualized treatment effect estimates may be helpful when discussing treatment options with a patient as this allows patients to make better-informed decisions. Shared decision-making in this context is already widely advocated by current guidelines [[1], [2], [3]]. This emphasizes the value of the VTE-PREDICT risk score to predict absolute increase in risk of clinically relevant bleeding and reduction in risk of recurrent VTE for individual patients.

Previous literature mainly focused on cutoff risk values to justify extended anticoagulation (eg, <5% annual risk of recurrent VTE [23] or cessation of treatment, without considering both risks simultaneously [24]). To our knowledge, ideal ratios between risks have not been studied before. The use of a decision analysis helps to consider not only fatal events but also the impact on quality of life. These results may be used to inform guidelines regarding treatment strategies for VTE and provide starting points for future studies on extended anticoagulation.

However, there are also some noteworthy limitations. First, treatment effect estimates were found to have a large impact, while these were based on a small number of studies. Estimates for individual DOACs are heterogeneous, but it is unclear to what extent this reflects actual differences in the effect or just differences in study design and population [25]. A head-to-head comparison is warranted to draw definitive conclusions on their comparative performance. Although reduced-dose DOACs are increasingly used for extended anticoagulation in clinical practice, treatment effect estimates of individual DOACs are especially heterogeneous, and robust pooled effect estimates are lacking. Hence, the base case analysis focused on full-dose DOAC. To inform clinical practice based on the best possible outcome, random discontinuation of treatment was not included in the base case analysis. Although the Bleeding Risk Study is a real-world study including patients who are, overall, representative of those in whom the decision must be made to stop or continue anticoagulation, some characteristics of the population may have influenced our results. The simulated population included a relatively high proportion of patients with unprovoked VTE, whereas those with bleeding risk factors (eg, history of cancer, liver disease, and alcohol abuse) were underrepresented. These characteristics may have led to a higher proportion of patients assigned to extended anticoagulation. Also, of note, only 36% of patients were female. Our approach shared the limitation of the VTE-PREDICT risk score, which excluded potentially relevant predictors of recurrent VTE and bleeding. This omission may have led to less heterogeneity in predicted risks. Furthermore, the comparison between VTE-PREDICT-guided management and current practice should be interpreted with caution for multiple reasons. The data did not allow us to differentiate between minor and major transient risk factors, while this distinction impacts recommended treatment. Moreover, the VTE-PREDICT risk score was used to simulate probabilities of recurrent VTE and bleeding in the microsimulation analyses. This may have led to overestimation of the clinical value of the VTE-PREDICT risk score but did not impact the comparison across ratios or the estimation of the optimal ratio. For computational reasons, incidence rates of recurrent VTE were assumed to be stable over time within the 5-year time horizon. However, in reality, rates may decrease over time. The VTE-PREDICT risk score was developed to predict first recurrent events and first clinically relevant bleeding events. In our simulation, patients could potentially have multiple events based on the same predicted risks. As the number of patients with recurring events is presumably small, the influence of this was assumed to be limited. Lastly, patients’ perspectives on recurrent VTE and clinically relevant bleeding are very heterogeneous. This is reflected by the wide confidence intervals of utility estimates. Our results underline the need for better studies on patient-reported outcomes and to define utilities more accurately for future studies. Assessing utilities in relevant subgroups may be helpful to increase precision. However, as can be appreciated from sensitivity analyses, using lower bound versus upper bound for utility estimates did not alter our conclusions.

## 5 Conclusions

A ratio between recurrence risk reduction and increase in risk of clinically relevant bleeding with extended treatment with full-dose DOAC of 0.90 (95% CI, 0.51-3.40; ie, a bleeding event is considered 0.90 the severity of a recurrent VTE) resulted in the highest number of QALYs within 5 years. However, this corresponds to a need to extend treatment in 99% of patients for a gain of less than 1 day in perfect health per year, yet without taking into account the burden associated with medication use. Hence, with the current evidence, no actual optimal weighing ratio to be used in clinical practice can be determined. Given the potential burden of treatment and the adage “First, do no harm,” one may question the clinical relevance of extended anticoagulation. Our results confirm that clinical equipoise exists regarding the decision to stop or continue anticoagulation after the initial treatment for VTE. This emphasizes the importance of shared decision-making and considering patients’ preferences.
