# Supplementary material for: Balancing risks of recurrent venous thromboembolism and bleeding with extended anticoagulation: a decision analysis
Source: Res Pract Thromb Haemost. 2023 Nov 26;8(1):102274. doi: 10.1016/j.rpth.2023.102274 (PMC10784302; doi:10.1016/j.rpth.2023.102274)
Supplement: Supplementary material [file mmc1.docx]

**SUPPLEMENTARY APPENDIX**

**Table of contents**

|  | **Page number** |
| --- | --- |
| 1. **Expanded methods** | 2 |
|  |  |
| 1. **Tables**   Table S1. Predictors and coefficients in the VTE-PREDICT risk score  Table S2. Derivation of pooled treatment effect estimates  Table S3. Prediction model for mortality  Table S4. Health states and utilities  Table S5. Model assumptions  Table S6. Scenario analyses  Table S7. Comparison of patients with and without extended anticoagulation according to the optimal ratio and standard of care | 5  6  7  8  9  11  12 |
| 1. **Figures**   Figure S1. Distribution of predicted risks of recurrent VTE and bleeding  Figure S2. Deterministic analysis using point estimates for all parameters  Figure S3. One-way sensitivity analyses  Figure S4. Convergence plot  Figure S5. Probabilistic scenario analysis | 13  14  15  16  17 |
|  |  |
| 1. **References** | 19 |

## Expanded methods

*Data preparation*

Prior to simulating the data, single imputation using predictive mean matching was used to impute sporadically missing values in the Bleeding Risk Study data (<1% for BMI, 1% for hemoglobin, 7% for estimated glomerular filtration rate (eGFR)). Data for the present analysis was simulated using the copula package (R Statistical Software). Based on a visual inspection of the distribution of the variables, a gamma distribution was assumed for hemoglobin and BMI, whereas a normal distribution was assumed for eGFR and age. A binomial distribution was used for all categorical variables. Variables not available in the Bleeding Risk Study data were sampled based on previous literature. The proportion of patients with an index VTE event associated with estrogen therapy was simulated based on the prevalence in combined data of the Canadian-based REVERSE I and REVERSE II studies. REVERSE I and REVERSE II were prospective cohort studies including patients with first unprovoked VTE(1,2). Systolic blood pressure was simulated based on values in the Canadian population, stratified by age and sex(3). As no population values were available for patients aged 79 years and over, values in the age group between 70 and 79 years were assumed to apply to older patients as well. Platelet count was simulated based on the mean and distribution in the population used to develop the VTE-PREDICT risk score(4). History of cancer was assumed to be absent in all patients as cancer in the previous 5 years was an exclusion criterion for the Bleeding Risk Study, and the proportion of patients with a history of cancer more than 5 years ago is assumed to be low(5). Similarly, liver disease, alcohol abuse and recent surgery and were assumed to be rare in the Bleeding Risk Study population and therefore assumed to be absent for all patients in the simulated dataset. Pregnancy as provoking factor was assumed to be absent for all patients given the proportion of female patients and age range of the study population.

*Estimating individual effect of extended anticoagulant treatment*

The VTE-PREDICT risk score was developed to predict up to 5-year risks of recurrent VTE and clinically relevant bleeding in patients without active malignancy who completed the initial anticoagulant treatment of a minimum of 3 months for VTE(4). Combined data of 3 trials and 2 cohorts were used for model derivation (n=15,141, mean ± standard deviation age 57.1±15.8 years, 41% female, 69% with unprovoked VTE, 49% receiving extended anticoagulation). The model consists of 2 complementary Fine & Gray competing risk-adjusted models with recurrent VTE and clinically relevant bleeding as respective events of interest and mortality due to other causes as competing event. Models were derived in patients with and without extended anticoagulation combined using offset terms to adjust for treatment status for a population representative of clinical practice. Predictors included in the models and effect sizes are shown in Supplementary Table 1. External validation in 4 cohorts and 1 randomized trial showed adequate predictive performance of the VTE-PREDICT risk score in different clinical settings. By combining absolute risks with hazard ratios (HRs) from clinical trials and cohorts, absolute recurrence risk reduction and increase in risk of bleeding with extended anticoagulation can be predicted for individual patients. The VTE-PREDICT risk score has been made available worldwide through an online calculator, available through http://vte-predict.com. Detailed description of the methodology and results of the VTE-PREDICT model has been published elsewhere(4).

For every individual patient in the study population, the VTE-PREDICT risk score was used to calculate the absolute recurrence risk reduction and increase in risk of bleeding with extended anticoagulation within 5 years. For the base case analysis, a pooled estimate for the effect of extended treatment with full dose DOAC was used (Supplementary Table 2).

*Model description*

A microsimulation model was developed to simulate outcomes of each scenario in terms of number of events (recurrence and bleeding) and all-cause mortality within 5 years (i.e. time horizon for which the VTE-PREDICT risk score was developed). The cycle length used was 3 months, as this is the minimum initial anticoagulant treatment duration(6,7). Possible states and transitions are illustrated in a model schematic in Figure 1. At the end of each cycle, patients could remain in the same health state or progress to a different health state. Transitions between health states are sampled based on the probabilities of transitioning to any of the states for an individual patient. Individual transition probabilities are calculated based on individual estimates of risks of recurrent VTE, bleeding and mortality and overall probabilities available from previous literature. Risks of recurrent VTE and bleeding for each patient were calculated using 5-year estimates from the VTE-PREDICT risk score. We used a Weibull model to estimate 5-year risk of mortality developed in the original Bleeding Risk Study data. Predictors for this model were selected based on available literature and included age, sex, body mass index (BMI), smoking status, PE as index event, index event provoked by surgery, trauma or immobilization, history of myocardial infarction, history of VTE, history of stroke, and estimated glomerular filtration rate (eGFR) (Supplementary Table 3)(8–10). Linear intrapolation was used to calculate probabilities for recurrent VTE, bleeding and mortality within one cycle length instead of 5-year probabilities. All patients started with a baseline health utility value sampled from health values of the general Canadian population, stratified by age and sex(11). For all acute events as well as for any subsequent chronic state, a disutility was added. By multiplying the utility associated with a health state and the time spent in this health state, QALYs were calculated, assuming health values to be multiplicative(12). All health states and utilities included in the model are shown in Supplementary Table 4. An overview of all other relevant model assumptions is provided in Supplementary Table 5. In correspondence with Canadian guidelines for health economic evaluations, all events were discounted at an annual rate of 1.5%(13).

## Table S1. Predictors and coefficients in the VTE-PREDICT risk score for recurrent venous thromboembolism and bleeding during anticoagulation

|  | **Predictor** | **Recurrent VTE**  sHR (95% CI) | **Bleeding**  sHR (95% CI) |
| --- | --- | --- | --- |
| **Demographics and physical examination** | Age (per decade) | 1.01 (0.97-1.06) | 1.05 (1.03-1.08) |
|  | Female sex | 0.86 (0.75-0.98) | 1.14 (1.05-1.24) |
|  | BMI | 1.00 (0.99-1.02) |  |
|  | Systolic blood pressure (per 10 mmHg) |  | 1.07 (1.03-1.10) |
| **Index event** | PE | 1.02 (0.89-1.18) | 1.07 (0.98-1.17) |
|  | Provoked by surgery, trauma or immobilization | 0.81 (0.68-0.98) |  |
|  | Provoked by estrogen therapy | 0.68 (0.47-1.00) |  |
| **Medical history** | History of cancer | 1.53 (1.14-2.06) | 2.48 (2.00-3.07) |
|  | History of VTE | 1.13 (0.97-1.32) |  |
|  | History of bleeding |  | 1.26 (1.11-1.44) |
|  | Stroke |  | 1.26 (1.08-1.46) |
| **Lab values** | Hb (g/dL) |  | 0.95 (0.93-0.97) |
| **Co-medication** | NSAIDs |  | 1.22 (1.08-1.38) |

*Abbreviations: Hb hemoglobin; PE pulmonary embolism; NSAID non-steroidal anti-inflammatory drugs; sHR subdistribution hazard ratio; VTE venous thromboembolism*

## Table S2. Derivation of pooled anticoagulant treatment effect estimates

|  | **Recurrent VTE** | **Bleeding** | **Reference** | **Methods** |
| --- | --- | --- | --- | --- |
|  | **RR (95% CI)** | **RR (95% CI)** |  |  |
| **Full dose DOAC** | 0.17 (0.11-0.26) | 2.72 (1.44-5.16) | Ebraheem et al.(14) and included original publications | In the pooled estimate in the meta-analysis by Ebraheem et al., the reduced dose of apixaban was included as well. Therefore, data provided in this meta-analysis and the original AMPLIFY-EXT publication were used to calculate pooled estimates without the reduced dose apixaban using a random-effects model. Moreover, to calculate pooled treatment effects for the combined outcome of CRNMB and major bleeding, CRNMB events were extracted from the original publications and added to a random effects model. |
| **Reduced dose DOAC** | 0.26 (0.14-0.46) | 1.19 (0.81-1.77) | Vasanthamohan et al.(15) | To be interpreted with caution: partly based on comparison between reduced dose DOAC and aspirin rather than reduced dose DOAC versus placebo alone. |

*Abbreviations: CI confidence interval; DOAC direct oral anticoagulant; VTE venous thromboembolism*

## Table S3. Prediction model for mortality (Weibull model)

|  | **Predictor** | **HR (95% CI)** |
| --- | --- | --- |
| **Demographics and physical examination** | Age (per year) | 0.94 (0.92-0.97) |
|  | Female sex | 0.74 (0.41-1.32) |
|  | BMI (kg/m^2^) | 1.04 (1.0-1.09) |
|  | Current smoker | 0.45 (0.21-0.98) |
| **Index event** | PE | 0.81 (0.51-1.28) |
|  | Provoked by surgery, trauma or immobilization | 0.45 (0.23-0.89) |
| **Medical history** | Myocardial infarction | 0.63 (0.32-1.26) |
|  | History of VTE | 0.81 (0.51-1.27) |
|  | Stroke | 0.50 (0.23-1.12) |
| **Lab values** | eGFR (ml/min) | 1.00 (1.00-1.02) |
|  |  |  |
|  | **Shape coefficient** | 0.18 |
|  | **Scale coefficient** | 12.59 |

*Abbreviations: BMI body mass index; eGFR estimated glomerular filtration rate; HR hazard ratio; PE pulmonary embolism*

## Table S4. Health states and utilities

| **Transition probabilities** | **Mean** | **SD/SE/95% CI** | **Reference** |
| --- | --- | --- | --- |
| Risk of recurrent VTE | Individual predicted 3-month risk | | VTE-PREDICT(4) |
| Case fatality rate of recurrent VTE without extended anticoagulation | 0.038 | 0.020-0.061 | Khan et al.(16) |
| Case fatality rate of recurrent VTE during extended anticoagulation | 0.049 | 0.021-0.087 | Khan et al.(17) |
| DVT (proportion of recurrent VTE events among patients without PE) | 0.756 | 0.698-0.989 | Prandoni et al.(18) |
| PE (proportion of recurrent VTE events among patients without PE) | 0.244 | 0.192-0.302 | Prandoni et al.(18) |
| DVT (proportion of recurrent VTE events among patients with PE) | 0.434 | 0.345-0.527 | Prandoni et al.(18) |
| PE (proportion of recurrent VTE events among patients with PE) | 0.566 | 0.473-0.655 | Prandoni et al.(18) |
| DVT and PE (proportion of recurrent VTE events) | 0.030 | n.a. | Khan et al. (16) |
| Severe PTS (5-year risk) | 0.059 | 0.044-0.077 | Kahn et al.(19) |
| CTEPH (2-year risk among PE patients) | 0.057 | 0.025-0.128 | Berghaus(20) |
| Risk of clinically relevant bleeding | Individual predicted 3-month risk | | VTE-PREDICT(4) |
| Fatal major bleeding while on DOAC | 0.097 | 0.032-0.192 | Khan et al. (21) |
| Proportion of bleeding events being CRNMB | 0.717 | 0.586-0.826 | Wells et al.(5) |
| ICH among major bleeding events | 0.10 | 0.05-0.20 | Linkins et al.(22) |
| Proportion of fatal ICH among ICH | 0.45 | 0.17-0.75 | Linkins et al.(22) |
| Mortality due to causes other than VTE or bleeding | Individual predicted 3-month risk | | Newly derived Weibull model |
| Ratio for increased risk of mortality in patients with CTEPH | 1.30 | 0.98-1.73 | Chin Chwan Ng(23) |
| Probability of discontinuing anticoagulant treatment after intracranial bleeding | 0.616 | 0.594-0.637 | Murthy et al.(24) |
| Probability of discontinuing anticoagulant treatment after major bleeding (non-ICH) | 0.290 | 0.266-0.314 | Little et al.(25) |
| Probability of discontinuing anticoagulant treatment after CRNMB | Index DVT: 11%  Index PE: 5% | n.a. | EINSTEIN-DVT(26) and EINSTEIN-PE(27); Heisen et al.(28) |
| Drug discontinuation rate (unrelated to death/VTE/bleeding), monthly ***** | 0.019 for DVT; 0.023 for PE |  | EINSTEIN-DVT(26) and EINSTEIN-PE(27) |
| **Utilities** | **Mean** | **SD/SE/95% CI** | **Reference** |
| Recurrent DVT (acute, 1 month) | 0.884 | 0.674-1.00 | Locadia et al.(29) |
| Severe PTS (chronic) | 0.863 | 0.695-1.00 | Locadia et al.(29) |
| Recurrent PE (acute, 1 month) | 0.663 | 0.379-0.905 | Locadia et al.(29) |
| CTEPH (chronic) | 0.560 | SD 0.29  SE 0.016 | Meads et al.(30) |
| CRNMB (utility decrement for one cycle duration) | 0.0049 | 0.003 | Sullivan et al.(31) |
| Major bleeding (non-ICH; acute disutility) | 0.684 | 0.516–0.905 | Locadia et al.(29) |
| ICH (acute) | 0.347 | 0.147–0.558 | Locadia et al.(29) |
| Post-ICH | 0.713 | 0.702–0.724 | Rivero-Aries et al.(32) |
| Disutility for being on anticoagulant treatment (DOAC) ***** | 0.99 | n.a. | Connells(33) |

** Used in scenario analysis only.*

*Abbreviations: CI confidence interval; CRNMB clinically relevant, non-major bleeding; CTEPH chronic thromboembolic pulmonary hypertension; DVT deep venous thrombosis; ICH intracranial hemorrhage; PE pulmonary embolism; PTS post thrombotic syndrome; SD standard deviation; SE standard error; VKA vitamin K antagonist; VTE venous thromboembolism*

## Table S5. Model assumptions

| **Assumption** | **Explanation** |
| --- | --- |
| *Model assumptions* | |
| Linearity of predicted risks | Predicted risks of recurrent VTE, clinically relevant bleeding (VTE-PREDICT risk score) and mortality (newly derived Weibull model) were assumed to be linear within the 5-year time horizon of predicted risks. Hence, linear intrapolation was used to calculate probabilities for recurrent VTE, bleeding and mortality within one cycle length (3 months). |
| Baseline values remain unchanged | Baseline health values, age and other variables were not updated throughout the 5-year simulation. |
| Distributions used for sampling of parameters in Monte Carlo simulations | The following distributions were assumed when sampling parameters in Monte Carlo simulations:   - A multivariate normal distribution was assumed for the mortality model; - A beta distribution was assumed for all transition probabilities, utilities and ratios; - For the VTE-PREDICT risk score, coefficients were sampled from a normal distribution only given the complexity of the underlying models; - Log transformations were applied if indicated. |
| Unknown distribution | If no data on distribution of a variable was provided in available literature (anticoagulant discontinuation rates), 1 standard deviation was assumed to be 30% of the mean value. |
| *Acute events and chronic complications* | |
| Utilities for acute events apply during one cycle only | In line with previous literature, utilities for most acute events (recurrent DVT and/or PE, major bleeding and ICH) were assumed to apply for 1 month; disutility for CRNMB was applied for one cycle. If DVT and PE occurred within the same cycle, the lowest utility (i.e. for PE) was applied. |
| Timeline of CTEPH and PTS | Both CTEPH and PTS were assumed to be chronic and can start in any cycle after a recurrent VTE event. CTEPH and PTS after the index VTE event were not taken into account in the present analysis. |
| Severe PTS | Only severe PTS was considered in the analyses as mild PTS has been found not to give any disutility(34). |
| Post ICH state | All patients with ICH were assumed to enter a chronic state after one state of acute ICH, resembling long term complications of ICH. |
| Risk of death in subgroups | For patients with CTEPH, a ratio was applied to reflect the increased risk of death in this subgroup of patients which is not yet included in the model. Higher risk of bleeding associated with other states is either included in the model for mortality (history of stroke, and PE as index event) or as a health state (fatal bleeding and fatal recurrence). |
| *Anticoagulant treatment* | |
| Burden of anticoagulant treatment | No disutility was applied for the use of anticoagulant treatment, as many patients consider anticoagulant treatment not to be very burdensome. |
| Discontinuation of anticoagulant treatment | Anticoagulant treatment was discontinued after a bleeding event in a proportion of patients according to previous literature (Supplementary Table 4). Treatment was assumed to be continued in all other situations in the base case analysis to allow for conclusions based on an optimal treatment strategy. It is assumed that patients remain as adherent to their anticoagulant treatment as participants in the trials for the duration of their follow-up. Patients with a history of cardiovascular disease without antiplatelet therapy were assumed to start antiplatelet therapy when stopping anticoagulation. In a scenario analysis, treatment was discontinued in a random proportion of patients to reflect actual clinical practice. |
| Restarting anticoagulant treatment | Patients were assumed to restart anticoagulant treatment after any recurrent VTE event and remain on treatment until treatment is discontinued after a bleeding event (base case analysis) or after random discontinuation (scenario analysis). |
| Anticoagulant treatment and mortality | Anticoagulant treatment was assumed not to influence mortality due to causes other than recurrent VTE or bleeding. |
| Stable relative treatment effect | It was assumed that relative treatment effects (i.e.*,* the hazard ratios derived from meta-analyses or trials) are equal for all patients for whom a treatment is recommended. This assumption was made because 1) subgroup analyses from trials and meta-analyses have not identified significant differences in relative treatment effects, and 2) because subgroup analyses are underpowered to detect treatment effect heterogeneity. |
| Constant treatment effect over time | It was assumed that the relative treatment effects from trials and meta-analyses remain constant over time during the time horizon for the VTE-PREDICT models. This was assumed because no trials with a 5-year follow-up after the initial treatment for VTE have been conducted, but from a pathophysiologic perspective, no differences in the effect are expected. |
| Additive treatment effects and harms of oral anticoagulants and antiplatelet therapy | It was assumed that benefits and harms of any oral anticoagulant in combination with antiplatelet therapy are independent of one another. |

*Abbreviations: CTEPH chronic thrombo-embolic pulmonary hypertension; DVT deep venous thrombosis; ICH intracranial hemorrhage; QALY quality adjusted life year; PE pulmonary embolism; PTS post thrombotic syndrome; VTE venous thromboembolism*

## Table S6. Scenario analyses

| **Scenarios** | **Description** |
| --- | --- |
| Reduced dose DOAC | All patients on extended anticoagulation are assumed to be on reduced dose DOAC instead of full dose DOAC. Treatment effects are shown in Supplementary Table 2.  A wider range of ratios was evaluated in this scenario, as ratios between 0.1 and 10 did not cover either no patients or all patients being on extended anticoagulation. |
| Random treatment discontinuation | A proportion of patients discontinues anticoagulant treatment for reasons other than after a bleeding event (e.g., because of side effects, patient’s preference or logistic reasons). Probabilities of discontinuing treatment are based on previous trials (Supplementary Table 4). |
| Add disutility for medication | Every cycle a patient is on anticoagulant treatment, their utility is multiplied by 0.99 to account for the burden of being on anticoagulant treatment (Supplementary Table 4). |
| Equal death rate for all patients | Instead of using the newly derived Weibull model to predict risk of death due to causes other than recurrent VTE or bleeding, a 5-year risk of death of 0.0222 is assumed for all patients(35). |

*Abbreviations: DOAC direct oral anticoagulant*

## Table S7. Comparison of patients with and without extended treatment according to the VTE-PREDICT risk score using the optimal ratio and according to standard of care. Data are shown as n (%) or mean (standard deviation).

|  | Extended anticoagulation according to both approaches | Extended anticoagulation with optimal ratio only | Extended anticoagulation with standard of care only | No extended anticoagulation according to both approaches |
| --- | --- | --- | --- | --- |
| n (%) | 4044 (40) | 5888 (59) | 0 (0) | 68 (1) |
| Female sex | 1203 (30) | 2284 (39) | n.a. | 67 (99) |
| Age (years) | 51.3 (9.7) | 66.2 (13.8) | n.a. | 69.7 (13.8) |
| **Medical history** | | | | |
| Prior stroke | 0 (0) | 296 (5) | n.a. | 11 (16) |
| History of bleeding | 0 (0) | 309 (5) | n.a. | 7 (10) |
| History of VTE | 1502 (37) | 2098 (36) | n.a. | 14 (21) |
| Atherosclerotic cardiovascular disease | 159 (4) | 342 (6) | n.a. | 10 (15) |
| **Index event** | | | | |
| PE with or without DVT | 1957 (48) | 2810 (48) | n.a. | 39 (57) |
| Provoked by estrogen therapy | 82 (2) | 488 (8) | n.a. | 31 (46) |
| Provoked by surgery, trauma or immobilization | 100 (3) | 463 (8) | n.a. | 8 (12) |
| **Physical examination and laboratory measurements** | | | | |
| BMI (kg/m^2^) | 31.2 (6.6) | 30.9 (6.8) | n.a. | 32.2 (7.2) |
| Systolic blood pressure (mmHg) | 114.8 (15) | 120.1 (16.8) | n.a. | 131.2 (16.5) |
| Hemoglobin (g/dL) | 14.7 (1.4) | 13.8 (1.5) | n.a. | 12.7 (1.0) |
| **Concomitant medication** | | | | |
| Antiplatelet therapy | 0 (0) | 482 (8) | n.a. | 62 (91) |
| NSAIDs | 125 (3) | 215 (4) | n.a. | 7 (10) |

*Abbreviations: BMI body mass index; DVT deep venous thrombosis; Hb hemoglobin; PE pulmonary embolism; NSAID non-steroidal anti-inflammatory drugs; VTE venous thromboembolism*

## Figure S1. Distribution of predicted risks of recurrent venous thromboembolism and clinically relevant bleeding

**
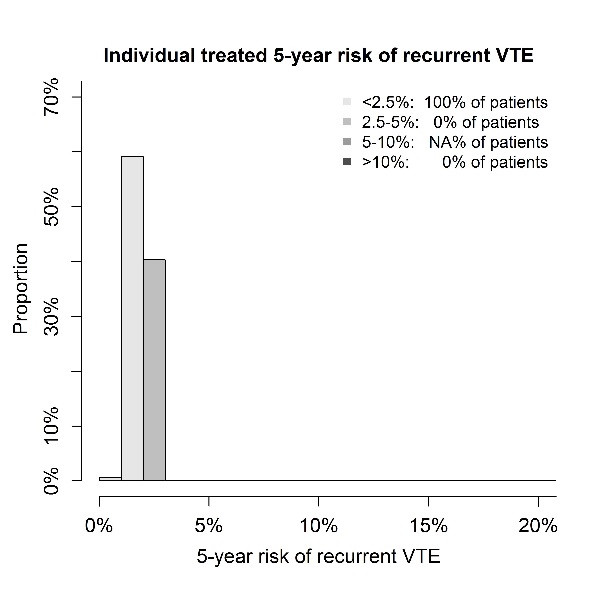

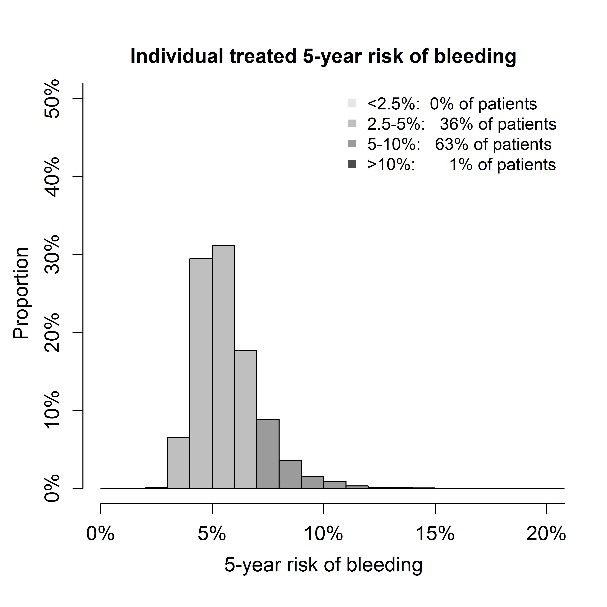

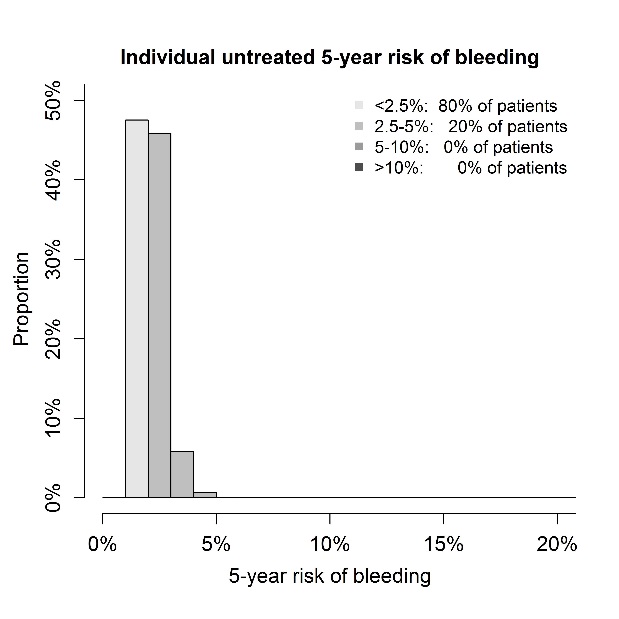

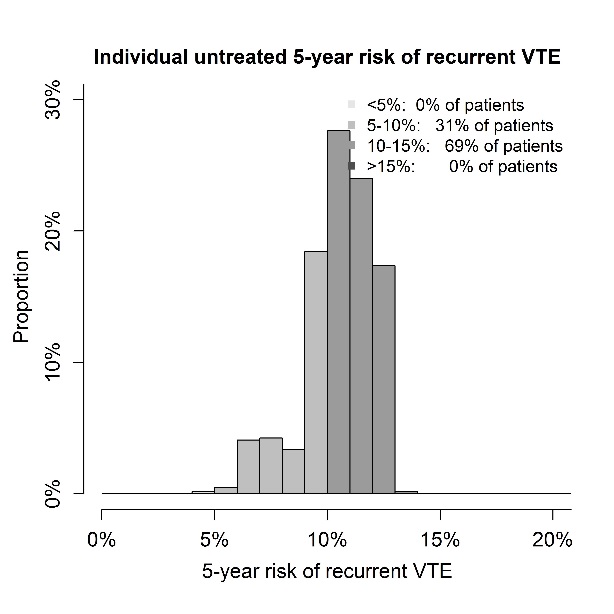
**

**
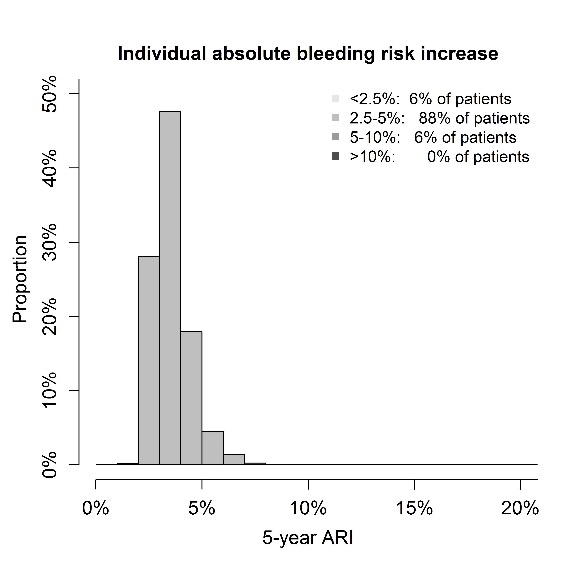

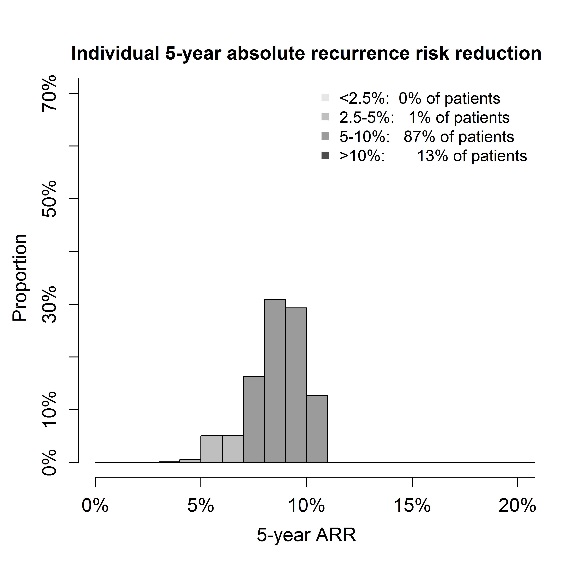
**

***Abbreviations:***

*Abbreviation: ARI absolute risk increase; ARR absolute risk reduction; VTE venous thromboembolism*

## Figure S2. Deterministic analysis using point estimates for all parameters of the base case analysis only


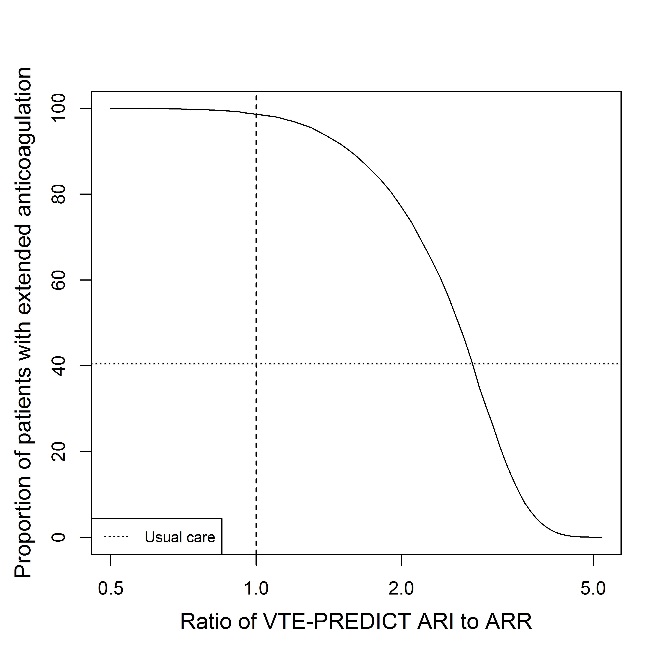

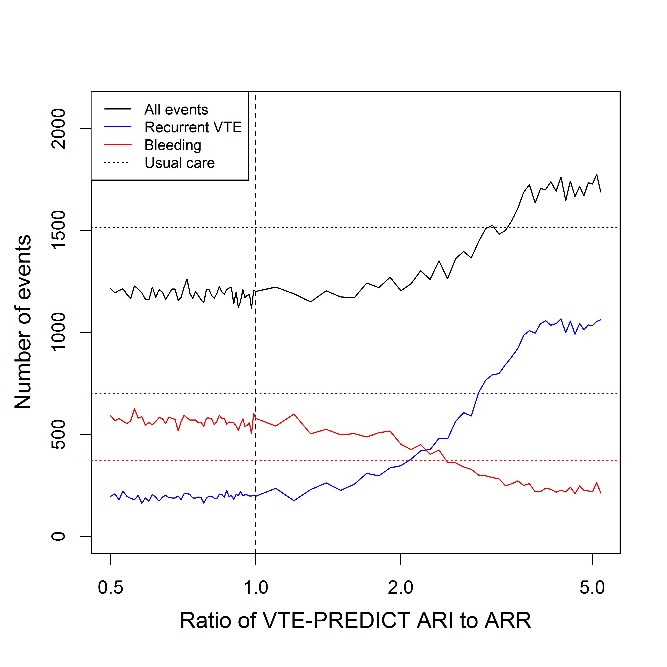

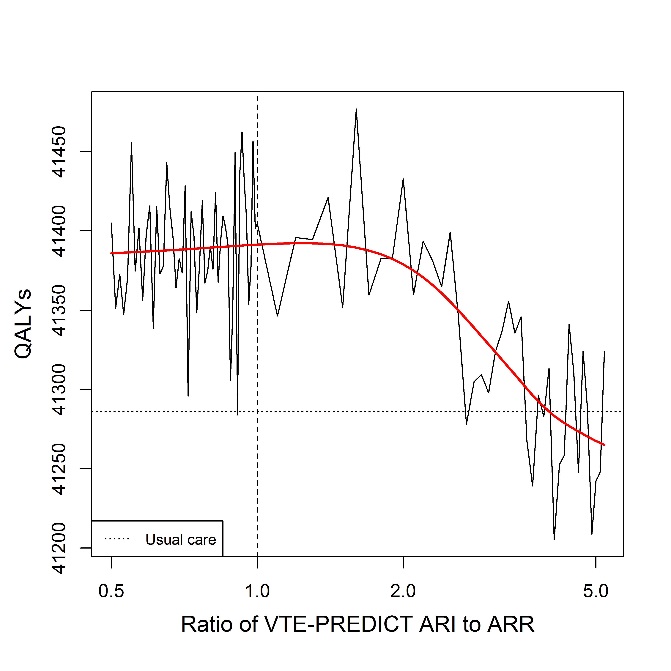


*Abbreviations: ARI absolute risk increase; ARR absolute risk reduction; QALY quality adjusted life year*

## Figure S3. One-way sensitivity analyses.

In each of these analyses, one parameter is changed at a time. Lower and upper confidence bounds are used. Results are to be compared to the results of the deterministic analysis for the base case.


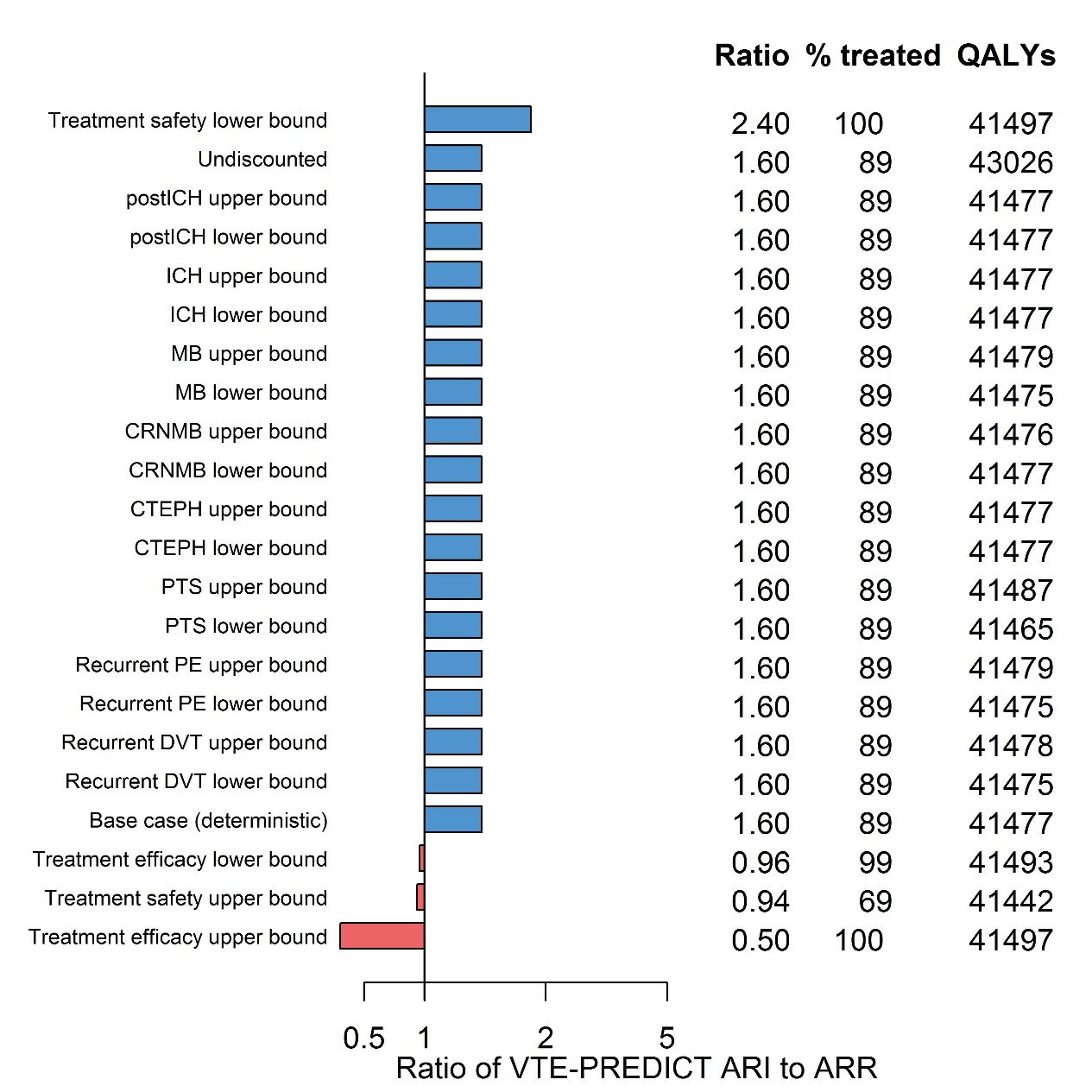


*Abbreviations: CTEPH chronic thromboembolic pulmonary hypertension; DVT deep venous thrombosis; ICH intracranial hemorrhage; PE pulmonary embolism; PTS post thrombotic syndrome; QALY quality adjusted life year*

## Figure S4. Convergence plot


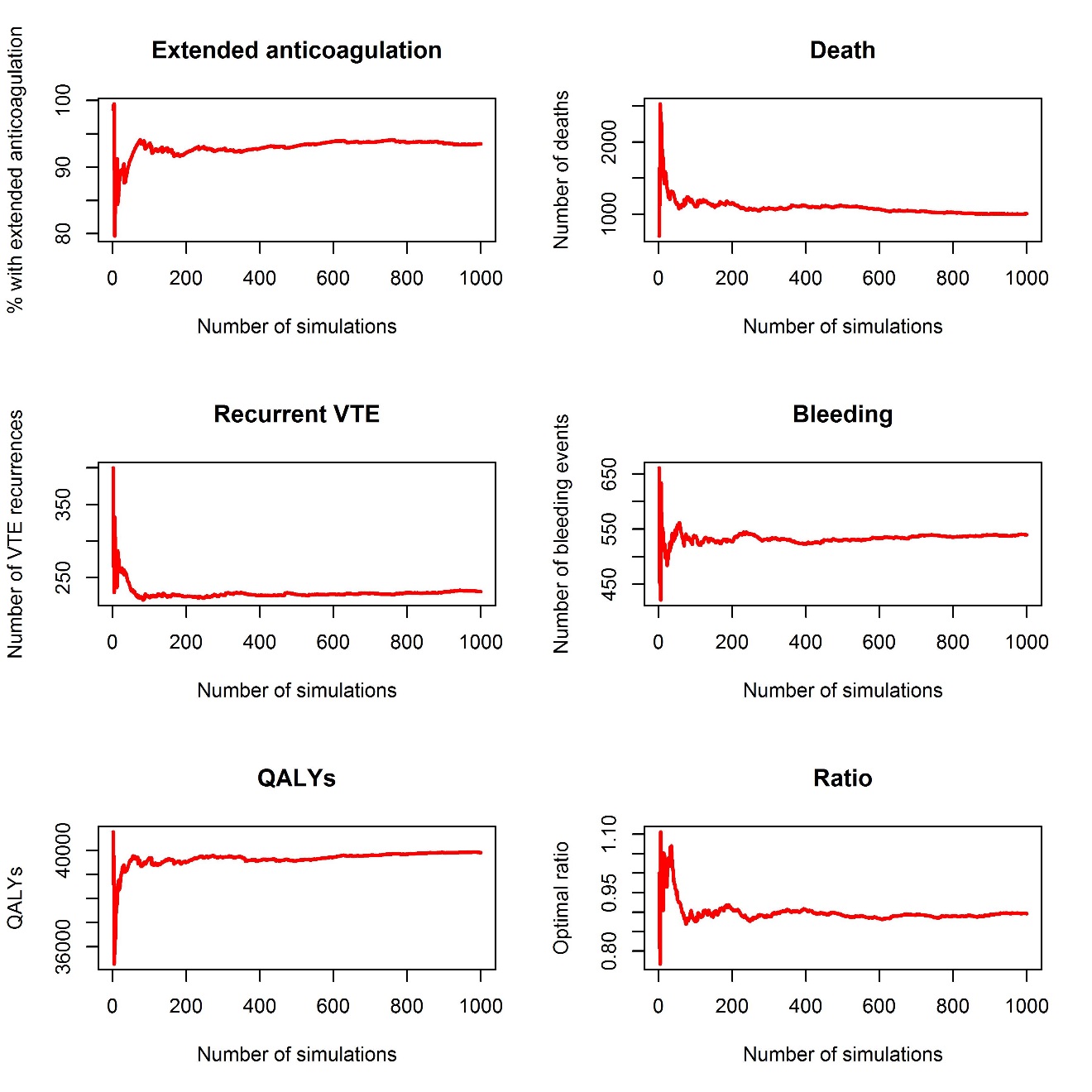


*Abbreviations: QALY quality adjusted life years; VTE venous thromboembolism*

## Figure S5. Probabilistic scenario analysis

1. Reduced dose DOAC


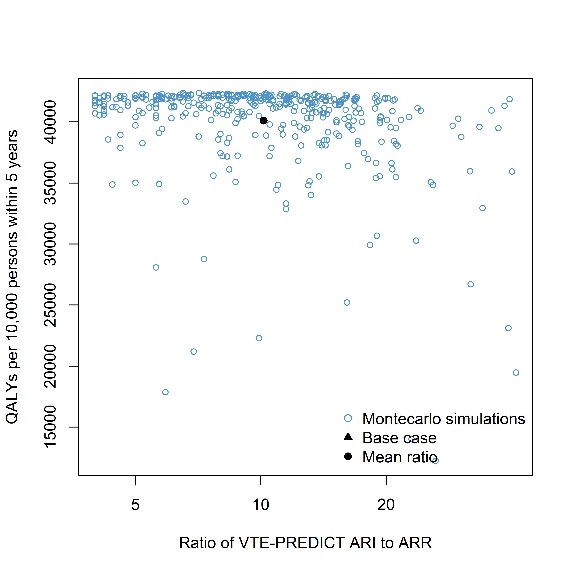

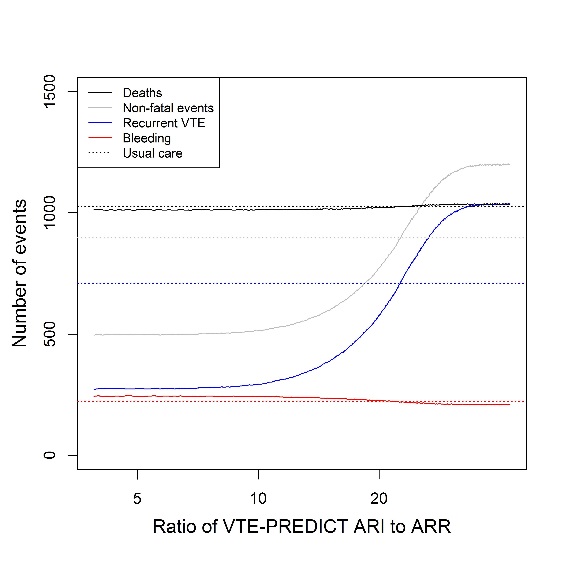

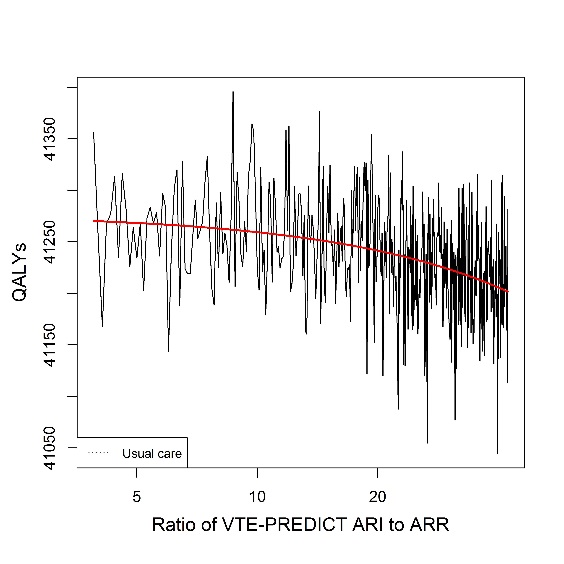


1. Random treatment discontinuation


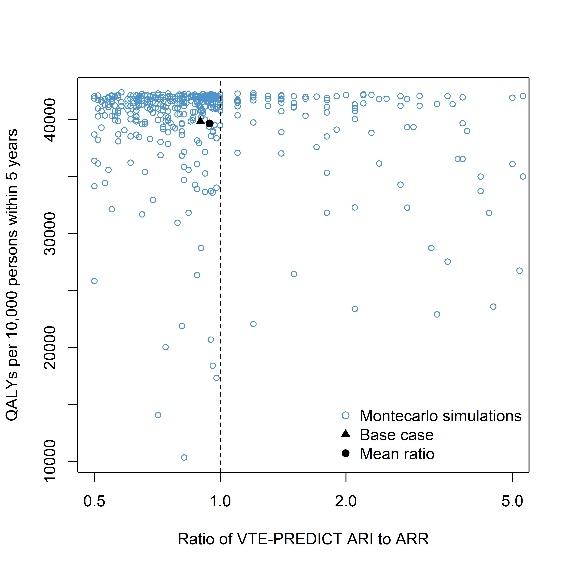

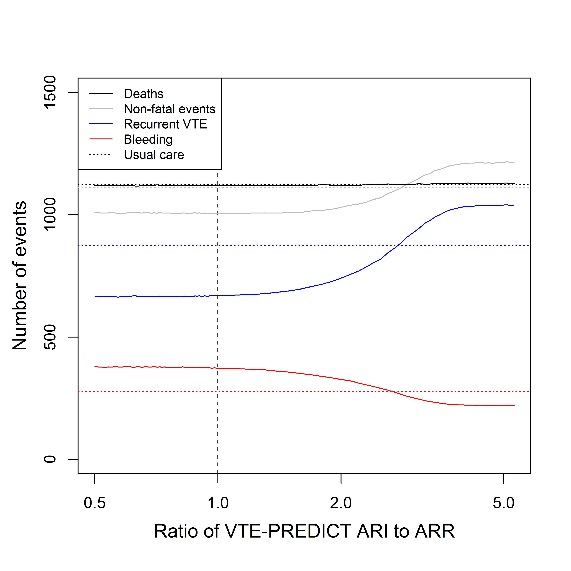

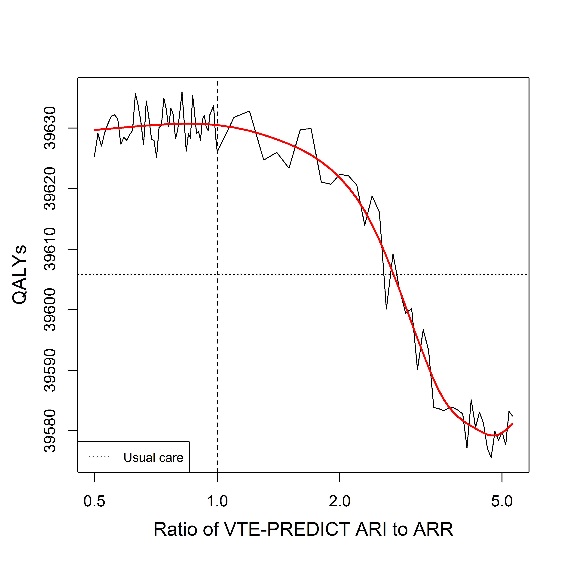


1. Add disutility for medication


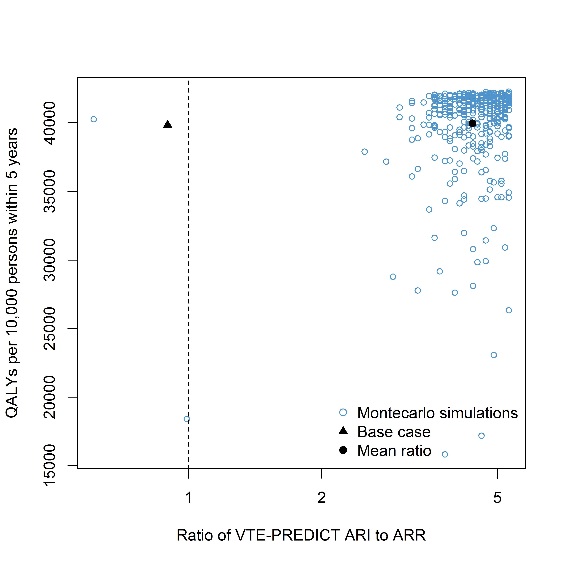

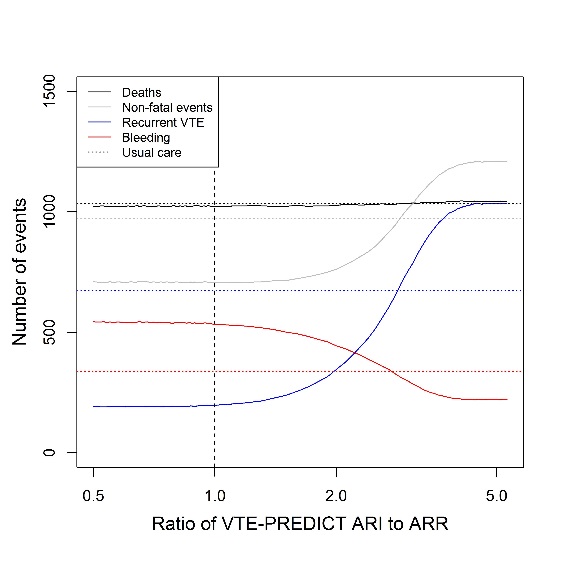

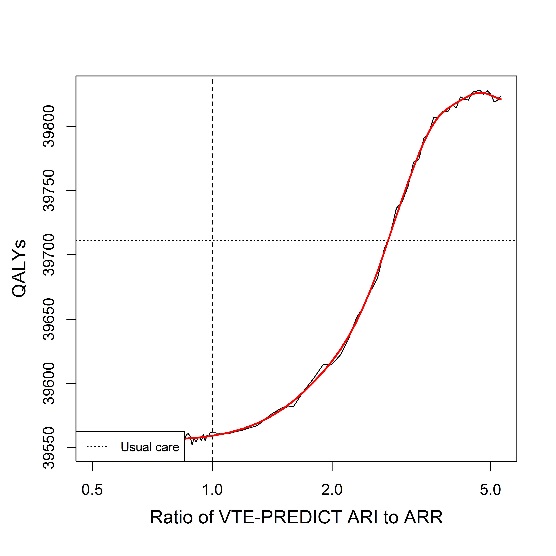


1. Equal death rate for all patients


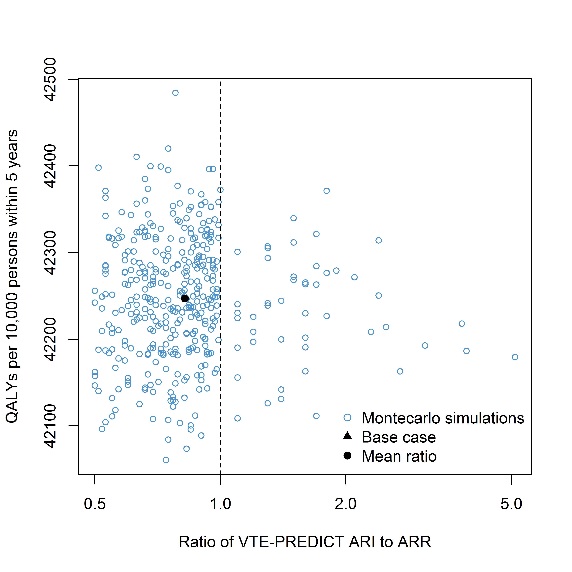

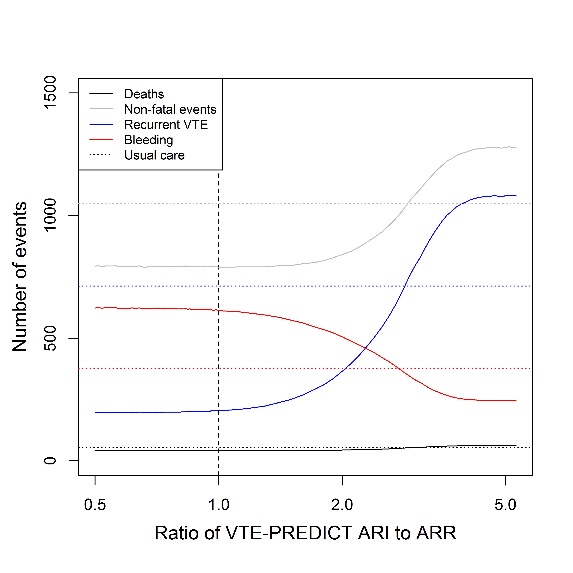

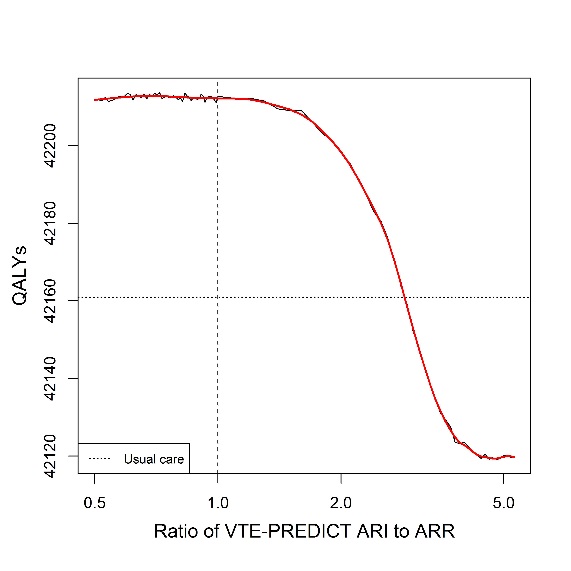


*Abbreviations: ARI absolute risk increase; ARR absolute risk reduction; QALY quality adjusted life year*

## References

1. Rodger MA, Le Gal G, Anderson DR, Schmidt J, Pernod G, Kahn SR, et al. Validating the HERDOO2 rule to guide treatment duration for women with unprovoked venous thrombosis: Multinational prospective cohort management study. BMJ Online. 2017;356.

2. Rodger MA, Kahn SR, Wells PS, Anderson DA, Chagnon I, Gal G Le, et al. for Recurrence Who Can Discontinue Anticoagulant Therapy. CMAJ. 2008;179(5).

3. DeGuire J, Clarke J, Rouleau K, Roy J, Bushnik T. Blood pressure and hypertension. Health Rep. 2019;30(2):14–21.

4. de Winter MA, Büller HR, Carrier M, Cohen AT, Hansen JB, Kaasjager KAH, et al. Recurrent venous thromboembolism and bleeding with extended anticoagulation: the VTE-PREDICT risk score. Eur Heart J. 2023 Jan 17;ehac776.

5. Wells PS, Tritschler T, Khan F, Anderson DR, Kahn SR, Lazo-Langner A, et al. Predicting major bleeding during extended anticoagulation for unprovoked or weakly provoked venous thromboembolism. Blood Adv. 2022;6(15):4605–16.

6. Boutitie F, Pinede L, Schulman S, Agnelli G, Raskob G, Julian J, et al. Influence of preceding length of anticoagulant treatment and initial presentation of venous thromboembolism on risk of recurrence after stopping treatment: analysis of individual participants’ data from seven trials. BMJ. 2011;342:d3036–d3036.

7. Konstantinides S V, Meyer G, Becattini C, Bueno H, Geersing GJ, Harjola VP, et al. 2019 ESC Guidelines for the diagnosis and management of acute pulmonary embolism developed in collaboration with the European Respiratory Society (ERS). Eur Heart J. 2019;ehz405.

8. Farjat AE, Fox KA, Turpie AG, Goldhaber SZ, Bounameaux H, Prandoni P, Weitz JI, Dalgaard F, Angchaisuksiri P, Haas S, Schellong S, Ageno W, Goto S, Panchenko E, Chiang C-, Jerjes Sanchez Diaz C, Muntaner J, Verhamme P, Tse E, Kakkar AK H. Prediction of Mortality in Patients with Recently Diagnosed Venous Thromboembolism: The GARFIELD-VTE Mortality Risk Model [abstract]. Res Pr Thromb Haemost. 2021;5 (Suppl 1).

9. Heit JA, Silverstein MD, Mohr DN, Petterson TM, O’Fallon WM, Melton LJ. Predictors of survival after deep vein thrombosis and pulmonary embolism: A population-based, cohort study. Arch Intern Med. 1999;159(5):445–53.

10. Næss IA, Christiansen SC, Romundstad P, Cannegieter SC, Rosendaal FR, Hammerstrøm J. Incidence and mortality of venous thrombosis: A population-based study. J Thromb Haemost. 2007;5(4):692–9.

11. Guertin JR, Feeny D, Tarride JE. Age- and sex-specific Canadian utility norms, based on the 2013-2014 Canadian Community Health Survey. Cmaj. 2018;190(6):E155–61.

12. Ara R, Brazier J. Estimating Health State Utility Values for Comorbidities. PharmacoEconomics. 2017;35:89–94.

13. McCabe C, Shinkins B, Lee K, LaPlante S. Guidelines for the Economic Evaluation of Health Technologies: Canada. CADTH METHODS AND GUIDELINES. 2019.

14. Ebraheem M, Alzahrani I, Crowther M, Rochwerg B, Almakadi M. Extended DOAC therapy in patients with VTE and potential risk of recurrence: A systematic review and meta-analysis. J Thromb Haemost. 2020;18(9):2308–17.

15. Vasanthamohan L, Boonyawat K, Chai-Adisaksopha C, Crowther M. Reduced-dose direct oral anticoagulants in the extended treatment of venous thromboembolism: a systematic review and meta-analysis. J Thromb Haemost. 2018;16(7):1288–95.

16. Khan F, Rahman A, Carrier M, Kearon C, Weitz JI, Schulman S, et al. Long term risk of symptomatic recurrent venous thromboembolism after discontinuation of anticoagulant treatment for first unprovoked venous thromboembolism event: systematic review and meta-analysis. BMJ. 2019;366:l4363.

17. Khan F, Tritschler T, Kimpton M, Wells PS, Kearon C, Weitz JI, et al. Long-term risk of recurrent venous thromboembolism among patients receiving extended oral anticoagulant therapy for first unprovoked venous thromboembolism: A systematic review and meta-analysis. J Thromb Haemost. 2021:1–13.

18. Prandoni P, Noventa F, Ghirarduzzi A, Pengo V, Bernardi E, Pesavento R, et al. The risk of recurrent venous thromboembolism after discontinuing anticoagulation in patients with acute proximal deep vein thrombosis or pulmonary embolism. A prospective cohort study in 1,626 patients. Haematologica. 2007;92(2):199–205.

19. Kahn SR, Shapiro S, Wells PS, Rodger MA, Kovacs MJ, Anderson DR, et al. Compression stockings to prevent post-thrombotic syndrome: a randomised placebo-controlled trial. Lancet Lond Engl. 2014;383(9920):880–8.

20. Berghaus TM, Barac M, von Scheidt W, Schwaiblmair M. Echocardiographic evaluation for pulmonary hypertension after recurrent pulmonary embolism. Thromb Res. 2011;128(6):e144-7.

21. Khan F, Tritschler T, Kimpton M, Wells PS, Kearon C, Weitz JI, et al. Long-Term Risk for Major Bleeding During Extended Oral Anticoagulant Therapy for First Unprovoked Venous Thromboembolism: A Systematic Review and Meta-analysis. Ann Intern Med. 2021;174(10):1420–9.

22. Linkins L, O’donnell M, Julian JA, Kearon C. Intracranial and fatal bleeding according to indication for long-term oral anticoagulant therapy. J Thromb Haemost. 2010;8(10):2201–7.

23. Chwan Ng AC, Chung T, Yong ASC, Wong HSP, Celermajer DS, Kritharides L. Long-term cardiovascular and noncardiovascular mortality of 1023 patients with confirmed acute pulmonary embolism. Circ Cardiovasc Qual Outcomes. 2011;4(1):122–8.

24. Murthy SB, Gupta A, Merkler AE, Navi BB, Mandava P, Iadecola C, et al. Restarting Anticoagulant Therapy After Intracranial Hemorrhage: A Systematic Review and Meta-Analysis. Stroke. 2017;48(6):1594–600.

25. Little DHW, Sutradhar R, Cerasuolo JO, Perez R, Douketis J, Holbrook A, et al. Rates of rebleeding, thrombosis and mortality associated with resumption of anticoagulant therapy after anticoagulant-related bleeding. CMAJ. 2021;193(9):E304–9.

26. Scott D, Brenner B, Buller HR, Gallus AS, Anthonie W, Misselwitz F, et al. Oral Rivaroxaban for Symptomatic Venous Thromboembolism. N Engl J Med. 2010;363(26):2499–510.

27. Büller HR, Prins MH, Lensin AWA, Decousus H, Jacobson BF, Minar E, et al. Oral rivaroxaban for the treatment of symptomatic pulmonary embolism. N Engl J Med. 2012;366(14):1287–97.

28. Heisen M, Treur MJ, Heemstra HE, Giesen EBW, Postma MJ. Cost-effectiveness analysis of rivaroxaban for treatment and secondary prevention of venous thromboembolism in the Netherlands. J Med Econ. 2017;20(8):813–24.

29. Locadia M, Bossuyt PMM, Stalmeier PFM, Sprangers MAG, van Dongen CJJ, Middeldorp S, et al. Treatment of venous thromboembolism with vitamin K antagonists: Patients’ health state valuations and treatment preferences. Thromb Haemost. 2004;92(6):1336–41.

30. Meads DM, McKenna SP, Doughty N, Das C, Gin-Sing W, Langley J, et al. The responsiveness and validity of the CAMPHOR Utility Index. Eur Respir J. 2008;32(6):1513–9.

31. Sullivan PW, Slejko JF, Sculpher MJ, Ghushchyan V. Catalogue of EQ-5D scores for the United Kingdom. Med Decis Mak Int J Soc Med Decis Mak. 2011;31(6):800–4.

32. Rivero-Arias O, Ouellet M, Gray A, Wolstenholme J, Rothwell PM, Luengo-Fernandez R. Mapping the modified rankin scale (mRS) measurement into the generic EuroQol (EQ-5D) health outcome. Med Decis Making. 2010;30(3):341–54.

33. Connell NT, Connors JM. Cost-effectiveness of edoxaban versus dalteparin for the treatment of cancer-associated thrombosis. J Thromb Thrombolysis. 2019;48(3):382–6.

34. Lenert LA, Soetikno RM. Automated Computer Interviews to Elicit Utilities: Potential Applications in the Treatment of Deep Venous Thrombosis. J Am Med Inform Assoc. 1997;4(1):49–56.

35. Nilius H, Mertins T, Boss R, Knuchel M, Blozik E, Kremer Hovinga JA, et al. Long-Term Survival After Venous Thromboembolism: A Prospective Cohort Study . Vol. 8, Frontiers in Cardiovascular Medicine. 2021.
